# Terrain park injuries and risk factors in western Canadian resorts, 2008–2009 to 2017–2018: insights for risk management

**DOI:** 10.3389/fspor.2024.1341265

**Published:** 2024-02-16

**Authors:** Tracey J. Dickson

**Affiliations:** University of Canberra Research Institute for Sport and Exercise (UC RISE), University of Canberra, Bruce, ACT, Australia

**Keywords:** terrain park, epidemiology, helmets, human factors, socioecological, skiing, snowboarding

## Abstract

Terrain parks (TP) are popular attractors to snowsport resorts for both skiers and snowboarders, however there is some concern about the risk of severe injury. TP risk management needs to balance the business case against the human cost of injury. To inform effective TP risk management strategies, it essential to understand risk factors, and injury frequency and severity. To this end, a retrospective inductive analysis of Canada West Ski Areas Association's Accident Analyzer database (2008–2009 to 2017–2018). Inclusion criteria., (i) at least 8 seasons of matching injury and participation data, (ii) minimum of 10 TP injuries p.a., (iii) activity either skiing or snowboarding, and (iv) injury location was coded as terrain park/rail. Data was excluded for ticket type N/A. Anonymised and deidentified secondary data was entered into SPSS for analysis. Between group differences were explored via *χ*^2^ analysis with Yates' Continuity Correction for 2 × 2 tables and an inductive data driven approach to explore other factors. From this data, 12,602 injuries were in TPs across 28 resorts. 11,940 (94.7%) met the inclusion criteria (14.2% female; 86.5% <25 years; 73.0% snowboarders. 50.8% were male snowboarders <25 years). Higher levels of helmet use were not correlated with a decline in reported head injuries. Day-ticket holders were more likely to be injured on their first two uses of a run than season pass holders. More snowboarders injured in TPs (59.7%) went to hospital than skiers (51.0%). Thus, participants injured in TP are typically younger, male, and snowboarders with either a Season Pass or day ticket, thus potentially a distinct target group for injury mitigation and prevention strategies and communications. The application of other frameworks such as the hierarchy of control and socioecological framework reflects the complex multifactorial systems in which snowsports occur and from which more targeted risk management strategies may emerge to mitigate injury risk while maintaining TP appeal.

## 1 Introduction

Monitoring injury trends is essential both to clarify the extent of the situation and to evaluate the efficacy of sport injury mitigation and prevention strategies. To develop evidence-based and data-driven risk management strategies, this research explores terrain park (TP) injuries and risk factors for alpine skiers and snowboarders in western Canadian resorts from 2008–2009 to 2017–2018, thus adding to previous research (1–3). Reflective of the interdisciplinarity of sport injury prevention (4) the discussion explores the results in light of several frameworks drawn from different disciplines and perspectives: hierarchy of controls, human factors, and the socioecological framework. These help to identify potential risk management strategies to address modifiable risk factors (5) such as who is most at risk of injury in TP within snowsport resorts that are complex, multifactorial environments (6).

TP are popular man-made features in snowsport resorts, part of their broader freestyle offerings (7). The importance of TP for the broader sport community is reflected in the inclusion of freestyle events like slopestyle and big air in World Cup and Olympic competition. Thus, TP are unlikely to be removed from resorts in the short term [despite some discussion to do so (1)], therefore it remains important to pursue research that explores efficient and effective ways to minimize the frequency and/or severity of TP injuries. It is also important to consider the effectiveness of injury mitigation, or prevention, strategies to inform future strategies. Despite TP popularity, research on injuries and risk management for injury prevention in TP has been limited and sporadic (8).

In the USA it was reported that 84% of TP injuries during 2000–2005 were snowboarders, where TP injuries were likely to be more severe than traditional slopes, often involving the head, though this research occurred when helmet use was less than 30% (9). Severity was defined as involving the head or spine or requiring transport to hospital. Austrian research (2010–2011) concluded that those most likely to be injured in TP are males and younger participants, of whom only 44% were snowboarders (10). A systematic review up to 2018 found that only two articles considered risk factors in TP, albeit the focus was upon feature design, and none addressed prevention strategies (8). They concluded that “no study examining prevention strategies in TPs or HPs (half-pipes) has been published and that the risk factors for injuries in TPs and HPs are understudied” (p. 23). Considering these findings, the following research explores risk factors and discusses potential prevention strategies in light of three systemic models that have been previously used in safety research as explored in the discussion.

The following explores data collected over a decade as part of a broader project (3, 11) to identify potential risk factors that can inform future injury prevention strategies considering these three models and frameworks.

## 2 Materials and methods

### 2.1 Databases

Across the decade 2008–2009 to 2017–2018, 10 seasons of data was collected about 133,781 snowsport injuries by western Canadian ski patrol departments from more than 50 member resorts. Details are recorded by ski patrollers using the National Ski Area Accident form. The deidentified anonymized data was voluntarily uploaded by resorts to the Canada West Ski Areas Association's (CWSAA) Accident Analyzer database.

The secondary anonymized deidentified data was included for further analysis from the data that was provided by CWSAA to the researchers if they met the five inclusion criteria,

• there was at least 8 seasons of matching injury and participation data,
• a minimum of 10 TP injuries p.a.,
• involving either alpine skiing or snowboarding, and for TP risk factors, iv) the injury location was coded as terrain park/rail (1,793 cases excluded).
• data was also excluded if the ticket type was N/A (*n* = 6,147).
• for analysis of injuries within TPs and for comparison with injuries outside the TP, data was included where the injury location was coded as terrain park/rail.

The use of secondary deidentified and anonymized data is consistent with Australia's National Statement on Ethical Conduct in Human Research 2007 (Updated 2018).

### 2.2 Instrument

The variables used from the National Ski Area Accident form were:

• Patient,
  ◦ Age (open ended, recoded here into two groups)
  ◦ Gender (male or female)
  ◦ Ticket type (six categories: day, multi-day, Season pass, other pass, N/A, unknown)
  ◦ Drugs/Alcohol (open ended, recoded into two categories)
• Complaint i.e., bodily injury zone (26 categories)
• Treatment protocol i.e., injury type (16 categories, including fracture, sprain, bruise, laceration, dislocation and concussion)
• Location:
  ◦ Incident location (12 categories, recoded into terrain park and other)
  ◦ Activity (8 categories of which only alpine skiing and snowboarding were used)
• Conditions:
  ◦ Helmet [three ownership categories (owned, rental, other) recoded into two categories]
  ◦ Ability, i.e., skill level (seven categories: beginner, novice, intermediate, advanced, expert, unknown, N/A),
  ◦ Transport: Destination from base (10 categories: private car, taxi, company, ambulance, bus, helicopter, walk/ski, other, unknown, N/A)

### 2.3 Analysis

Injury rates were calculated in comparison to matching participation data, also known as skier visits. There is no data that differentiates between time in TP vs. time elsewhere in the resort. Between group differences and risks were explored through *χ*^2^ analysis and odds ratios that was informed by previously identified risk factors and outcomes in snowsport injuries including, nominated gender (female vs. male) (9, 10, 12), age (≤25 years vs. >25 years plus) (7, 9, 10), snowsport activity (alpine skiing vs. snowboarding) (12), helmet use (Yes or No) (12), self-assessed skill level (Beginner, Novice, Intermediate, Advanced, Expert) (2, 7, 9), drug and alcohol use considered a contributing factor (Yes/No or N/A) (13), and non-facial head injury type and severity (concussion, bruise, laceration, fracture, other) (9, 14), and injury severity using the proxy of destination from base (return to activity, home, hotel or rest, clinic or doctor, hospital) (9, 14) (Table 1). Where there were significant differences, Bonferroni *post hoc* tests were conducted. An inductive, data-driven approach was used to explore other possible risk factors, such as ticket type and frequency of run use of, and, given helmet (a form of PPE) usage rates, the main head injury types were explored. Cases were excluded from analysis where relevant data is missing.

**Table 1:** Between group differences for alpine skiers and snowboarders injured in terrain parks compared to injuries on all other lift-accessed terrain.

		Injuries in terrain park *n* = 11,690	Injuries in all other lift-accessed resort areas *n* = 87,910	All injuries *n* = 99.600	*χ* ^2^ * *p < .001*	*df*	*Effect size: φ or Cramer's V (Weak if <0.4)*	Post-hoc analysis with Bonferroni correction ** = Significant	Odds ratio (95% CI)
Activity type	Alpine skiing	27.1%	52.2%	49.3%	2,593.9*	1	.161		2.93 (2.81–3.01)
	Snowboarding	72.9%	47.8%	50.7%					
Gender	Female	14.3%	45.5%	41.8%	3,928.9*	1	.203		4.99 (4.72–5.27)
	Male	85.7%	54.5%	58.2%					
	Missing data			4.487					
Age group	≤25 years	86.4%	63.6%	66.3%	2,372.9*	1	.156		.27 (.26–.29)
	>25 years	13.6%	36.4%	33.7%					
	Missing data			1,436					
Ticket type	Day ticket	42.2%	56.3%	54.7%	2,242.3*	3	.150	**	
	Multi-day	7.8%	14.7%	13.9%				**	
	Season pass	46.9%	26.4%	28.8%				**	
	Membership/other	3.1%	2.5%	2.6%					
Self-reported ability level	Beginner	2.9%	29.7%	26.6%	6,410.4*	4	.257	**	
	Novice	6.8%	15.1%	14.1%				**	
	Intermediate	35.4%	30.1%	30.8%				**	
	Advanced	39.6%	18.0%	20.5%				**	
	Expert	15.3%	7.1%	8.0%				**	
	Missing data			2,823					
Helmet use All	Yes	84.0%	80.2%	80.7%	93.5*	1	.031		1.03 (1.02–1.04)
Alpine skiers		92.1%	83.5%	84.1%					
Snowboarders		81.0%	76.6%	77.3%					
	Missing data			3,709					
Head injury type (4 main)	Concussion	11.5%	8.6%	8.9%	800.8*	4	.090	**	
	Bruise	8.8%	8.6%	8.6%					
	Laceration	5.7%	5.2%	5.3%				**	
	Fracture	37.3%	27.8%	28.9%				**	
	Other	36.6%	49.8%	48.2%				**	
Destination from ski patrol base (proxy for severity)	Return to activity	4.3%	4.0%	4.0%	403.0*	3	.065		
	Home, hotel, or rest	27.2%	36.2%	35.1%				**	
	Clinic or doctor	11.0%	11.7%	11.6%				**	
	Hospital	57.5%	48.2%	49.3%				**	
	Missing data			4,752					
Drug and/or alcohol contributed to injury event	Yes	1.8%	0.9%	1.0%	60.8*	1	.032		1.14 (1.09–1.19)
	Missing data			40,083					

* *p*<.001 (*χ*^2^ test).

** significant (Bonferroni test).

## 3 Results

From the original dataset of 133,781 cases, 99,600 (74.4%) were retained that met the five inclusion criteria. Of these, 11,940 (12.0%) were injuries to alpine skiers and snowboarders in TPs were across 19 resorts (14.2% female; 86.5% <25 years of age; 73.0% snowboarders) (Table 1). The average injury rate for TP injuries by alpine skiers and snowboarders per 1,000 skier visits, calculated from matching the whole-of-resort participation data, was 0.30, ranging from .14 to .34. This equates to 3,301 mean days between injury (MDBI).

### 3.1 Injuries in terrain parks compared to other lift-accessed resort areas

*χ*^2^ analyses indicated significant differences in all the following nine comparisons between those injured in TP vs. those injured in other lift-accessed areas, however with only small or weak effect sizes (see Table 1). Where there were significant differences, Bonferroni *post hoc* analyses were also conducted for data in larger than 2 × 2 tables.

For nominated gender, proportionately less females were injured in TPs (14.2% c.f. 45.1%) than males (85.8% c.f. 54.9%). With respect to age, more younger participants, <25 years, were injured in TPs vs. other areas (86.5% c.f. 63.8%) than older participants (13.5% c.f. 36.2%). Regarding ticket type, in TP, season-pass holders were the largest injured group compared to non-TP injuries (46.8% c.f. 26.6%), followed by day-ticket holders (42.2% c.f. 56.2%). For self-reported ability or skill levels, in TP, significantly more intermediate and advanced participants were injured (75.0%) while in other areas it was more likely to be beginner, novice and intermediate participants (74.9%).

Helmet wearing was higher by those injured in TP (84.0%) compared to those injured outside TP (80.2%). Given this difference in helmet usage, differences in the main types of head injuries [i.e., concussion bruise, laceration, and fracture (11)] were also explored. Facial injuries were excluded as recreational helmets are not designed to protect the face. Head injuries in TP were significantly different from those outside, with TP having a higher proportion of all four head injury types (Table 1).

With using destination from the ski patrol base as a proxy for injury severity, significantly, more TP injuries (57.5%) than non-TP injuries (48.2%) were taken to hospital. Patrollers reported that drugs and alcohol were involved in twice as many TP injuries (1.8%) as non-TP injuries (0.9%).

Bonferroni *post hoc* analyses revealed significant differences at *p < .*05 for: all ticket types, except membership or other; all ability levels; four head injury types; and all destinations from base, except return to activity.

### 3.2 Injuries in terrain parks (*n* = 11,690)

*χ*^2^ analysis was used to compare the two main activity groups participating in TP, alpine skiing and snowboarding (see Table 2). Where results were significant, there was only a small or weak effect size. Where there was a significant difference, Bonferroni *post hoc* analyses at *p *< .05 were conducted where data was in larger than 2 × 2 tables.

**Table 2:** Between group differences for alpine skiers and snowboarders injured in terrain parks.

		Alpine skiers injured in terrain parks *n* = 3,173	Snowboarders injured in terrain parks *n* = 8,517	All TP injuries *n* = 11,690	*χ*^2^ *p < .001	*df*	*Effect size: phi or Cramer's V (Weak if <.4)*	Post-hoc analysis with bonferroni correction ** Significant	Odds ratio (95% CI)
Gender	Female	13.9%	15.3%	14.3%	3.34	1	.018		1.12 (.99–1.26)
	Male	86.1%	84.7%	85.7%					
	Missing data			515					
Age group	≤25 years	88.4%	85.7%		13.70*	1	.156		1.27 (1.12–1.44)
	>25 years	11.6%	14.3%					**	
	Missing data			168					
Ticket type	Day ticket	36.0%	44.5%		90.80*	3	.088	**	
	Multi-day	10.5%	6.8%					**	
	Season pass	50.4%	45.6%					**	
	Membership/other	3.2%	3.0%						
Self-reported ability level	Beginner	3.1%	2.8%	2.9%	278.92*	4	.157		
	Novice	6.7%	6.8%	6.8%					
	Intermediate	27.0%	38.6%	35.4%				**	
	Advanced	39.4%	39.6%	39.6%					
	Expert	23.8%	12.2%	15.4%				**	
	Missing data			393					
Helmet use	Yes	92.1%	81.0%	84.0%	203.83*	1	.031		
All									
	Missing data			386					
Head injury type (4 main)	Concussion	13.6%	10.8%	11.5%	110.00*	4	.097	**	
	Bruise	10.7%	8.1%	8.8%				**	
	Laceration	6.7%	5.4%	5.7%				**	
	Fracture	30.1%	40.0%	37.3%				**	
	Other	38.9%	35.7%	36.6%				**	
Destination from ski patrol base (proxy for severity)	Return to activity	5.2%	3.9%	4.3%	85.85*	3	.088	**	
	Home, hotel, or rest	32.9%	25.1%	27.2%				**	
	Clinic or doctor	10.6%	11.2%	11.0%					
	Hospital	51.3%	59.8%	75.5%				**	
	Missing data			545					
Drug and/or alcohol contributed to injury event	Yes	0.8%	2.2%	1.8%	15.01*	1	.046	**	2.85 (1.66–4.89)
	Missing data								
Main injury zones	Lower limb	30.8%	15.9%	19.9%	477.94*	4	.202	**	
	Upper limb	33.3%	52.0%	47.0%				**	
	Abdomen and back	13.9%	15.7%	15.2%				**	
	Head and face	20.2%	14.7%	16.2%				**	
	Other incl medical	1.9%	1.6%	1.7%					

* *p*<.001 (*χ*^2^ test).

** significant (Bonferroni test).

There was no significant difference between the activity and gender, with proportionately less females injured in TP in both skiing and snowboarding (15.3% and 13.9% respectively), *p *= .063. There was a significant difference between younger vs. older participants in skiing (88.4% c.f. 11.6%) and snowboarding (14.3% c.f. 85.7%). The significant difference in ticket-types revealed that more skiers used a season pass than snowboarders (40.4% c.f. 45.6%), while more injured skiers were using a day ticket than skiers (44.5% c.f. 36.0%). For ability level, the *χ*^2^ analysis revealed significant difference between skiers and snowboarders. More advanced and expert skiers were injured (39.4% and 23.8%) while more snowboarders were intermediate and advanced (38.6% and 39.6%).

Helmet wearing by injured participants in TP increased from 72.9% in 2008–2009 to 92.0% in 2017–2018 *χ*^2^ (1, *n *= 11,304) = 316.78, *p *< .001, *φ *= .17, while the mean reported head injuries of TP injuries across the decade was 11.1% (SD = 3.14). There was a significant difference between the proportion of reported injuries that were to the head between helmet and non-helmet wearers (11.4% c.f. 9.6% respectively), *χ*^2^ (1, *n *= 10,792) = 4.79, *p = *.028, *φ *= .02. There were also significant differences for helmet wearing between alpine skiers injured in TP (92.1%) and snowboarders injured in TP (81.0%). There was no significant difference in helmet wearing across skill levels, beginner (81.2%), novice (86.9%), intermediate (83.8%), advanced (83.8%) and expert (84.4%), *χ*^2^ (4, *n *= 10,972) = 6.97, *p = *.138.

Significantly, more day ticket holders and muti-day ticket holders (57.3% and 61.7% respectively) had only ridden that TP run only 1 or 2 times before their injury, compared to 30.8% of season pass holders (Table 3).

**Table 3:** Differences in ticket-type of those injured in terrain parks and other lift accessed terrain.

	Terrain Park Injuries						Injuries in other areas					
Ticket type	1st time on run	2nd time	3rd or later	*χ*^2^ *p < .001	*df*	Post hoc comparisons ** Significant	1st time on run	2nd time	3rd or later	*χ*^2^ *p < .001	*df*	Post hoc comparisons ** Significant
Day ticket	29.2%	27.8%	42.7%	828.97*	6	** all	44.0%	26.9%	29.1%	7,362.43*	6	** all
Multi-day	29.8%	31.3%	38.8%			** 2 vs. 3+	41.9%	28.1%	30.0%			** 2 vs. 3+
Season pass	13.8%	16.7%	69.2%			** all	20.2%	18.4%	61.4%			** all
Membership & other	19.2%	20.1%	60.5%				31.5%	21.2%	47.3%			** 2 vs. 3+

* *p*<.001 (*χ*^2^ test).

** significant (Bonferroni test).

In TP, snowboarders had significantly more upper limb injuries (clavicle to fingers) than skiers (52.0% c.f. 33.3%), while skiers had more lower limb injuries (foot to hip) than snowboarders (30.8% c.f. 15.9%). The main body part injured for skiers was the knee (13.6%) followed by the head (13.4%), while for snowboarders it was the wrist (18.9%) followed by the shoulder (15.4%). Skiers reported a higher rate of head injuries than snowboarders (13.4% c.f. 10.4%).

Of all TP-injuries, there were significant differences between activity and destination with more snowboarders (59.8%) going to hospital than skiers (51.3%). 56.0% of TP injuries transported to hospital were male snowboarders <25 years of age. There was a significant difference in destination from base across skill levels, although with a small effect size with intermediate, advanced, and experts requiring additional medical treatment from a clinic/doctor or hospital (68.3%, 68.8% and 69/9% respectively) compared to beginners (64.4%), and novices (64.2%), *χ*^2^ (12, *n *= 10,784) = 42.58, *p *< .001, *Cramer's V *= .04. Of the 11,690 TP incidents, 51.0% involved male snowboarders less than 26 years of age who were at greater risk of injury in a TP than outside, *χ*^2^ (1, *n *= 95.790) = 4,865.0, *p *< .001, *φ *= .23 (OR: 3.96, 95% CI: 3.80–4.12).

Bonferroni *post hoc* analyses were also conducted where significant differences were found in tables great than 2 × 2. Significant differences at *p* < .05 were found in: all ticket types, expect for membership; for intermediate and expert skill levels; for the four main head injury types. For destination from base, significant differences were demonstrated for all but clinic or doctor. All four main bodily injury zones were significantly different.

## 4 Discussion

Three systemic models are used to guide the discussion of the results. These models have been used in a variety of safety and participation research as indicated in the exploration of the models below.

### 4.1 Three systemic models

Firstly, one lens or framework to address injury reduction, from the broader field of occupational safety is the hierarchy of controls (HoC) that originated from the National Safety Council in the 1950s (15). Over time, there have been many variations suggesting it may be considered more a rule of thumb than a law or a theory (15). Current variations include four to six layers. Informed by a hazard assessment, common layers of the HoC are elimination (e.g., physically remove the hazard); substitute or replace the hazard; engineering controls (e.g., isolate people from the hazard); administrative controls (e.g., training or signs to change how people work), and personal protective equipment (PPE) (15–17). Having emerged from occupational safety the HoC focused upon the risks within a workplace or enterprise where it may be easier to monitor and manage workers' behaviors than in a leisure or recreational context. As snowsport resorts are workplaces, the HoC may be relevant especially where visitors to workplaces are included in the jurisdiction's work, health and safety legislation such as in Australia (18), or the USA where the mission of the Office of Occupational Safety and Health's is said to ensure the health, safety, and well-being of our employees and visitors' (italics added) (19). Further, workers such as instructors and TP staff, or crew, participate in, instruct and maintain TPs, and are thus at risk of injury within the TP.

**Figure 1:**
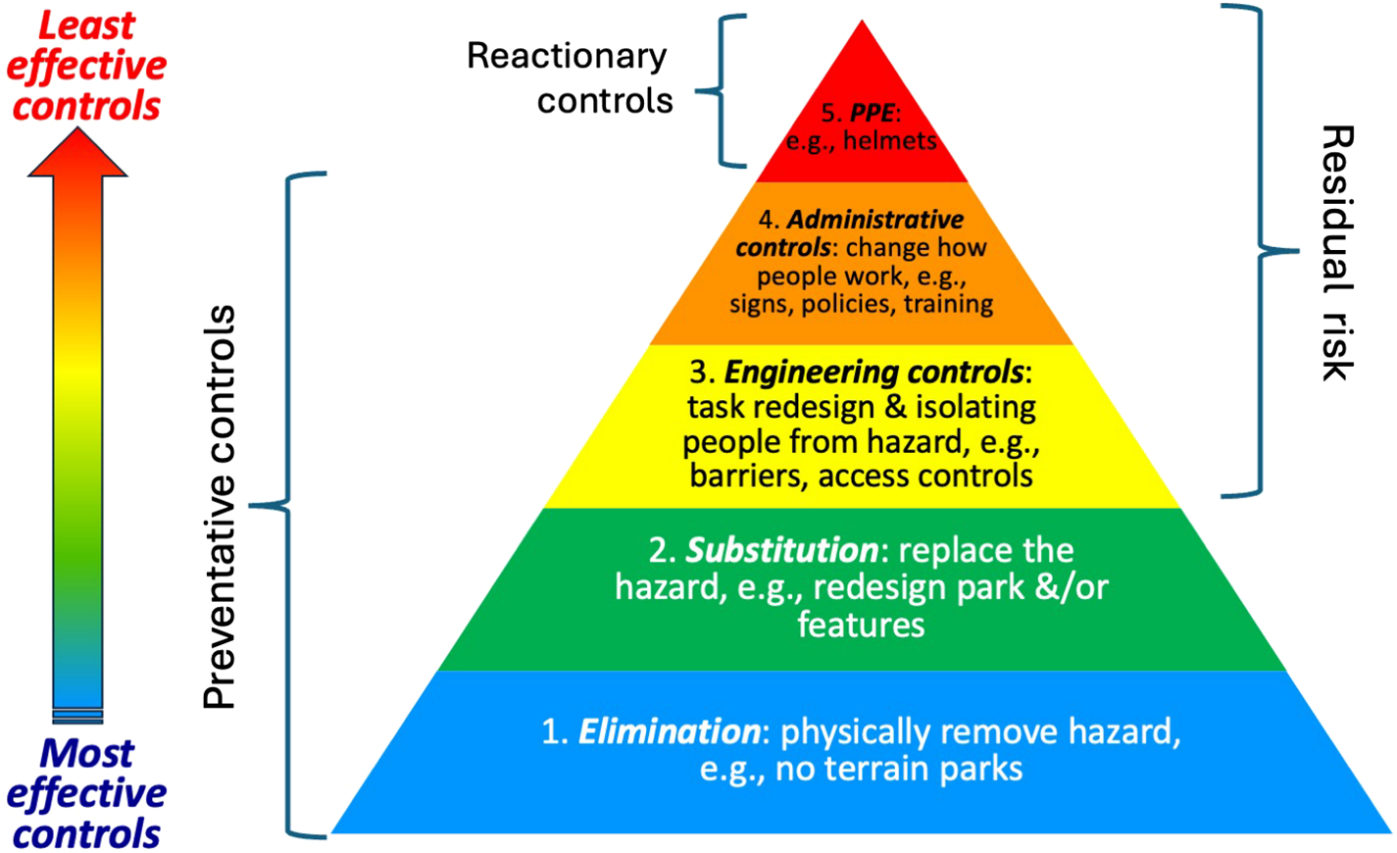
Hierarchy of controls adapted from Lyon and Popov (17) and CDC and NIOSH (16).

Recommendations from the extant TP safety research may be linked to a common variation of HoC: elimination, e.g., remove all terrain parks (1); substitution e.g., park redesign and equivalent fall from height? (14, 20, 21); isolation e.g., barriers to who access (2); administrative controls, e.g., policies, signs and training (2, 14, 21); and personal protective equipment (PPE), e.g., helmets (14, 22). However, a cautionary note from the safety literature (17) is that the most effective controls are the preventative ones from elimination to administrative, whilst the least effective is the reactionary use of PPE (Figure 1). If TPs are removed, it still may be tempting for participants to create their own jumps elsewhere in the resort, creating a different risk management issue.

Secondly, human factors (HF) provides a broader perspective where HF is considered a “scientific discipline concerned with the understanding of interactions among humans and other elements of a system, and the profession that applies theory, principles, data and methods to design, in order to optimize human well-being and overall system performance” (cited in 4) For TP, the complex multifactorial HF “system”, or environments, that TP users interact with include,

• natural: slope angles, weather, and snow conditions;
• built: terrain park design and maintenance, feature size, information, and signage;
• social: who is participating, age, gender, activity, skill level, motivations, and equipment choices e.g., PPE (4).

**Figure 2:**
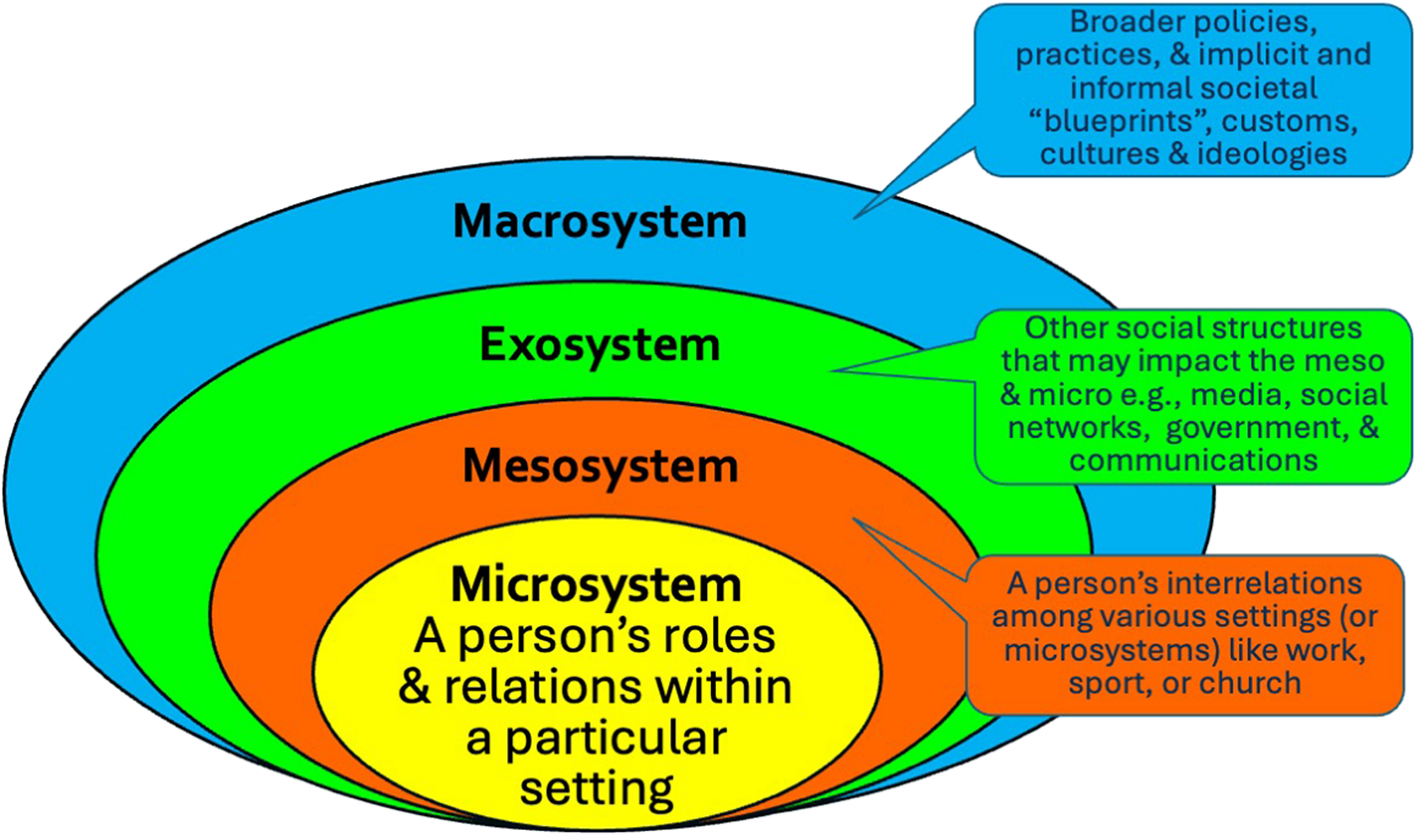
Socioecological framework adapted from Bronfenbrenner's 1977 ecology of human development.

Thirdly, in sport and public health, ecological models have been used to address “people's interactions with their physical and sociocultural surroundings” (23, p. 299) that is relevant to TP injury prevention. Like HF, ecological models include a wide array of influences upon behaviour, but in this case, they are considered in layers, much like an onion. Different models use different labels such as micro, meso, exo, and macro, and intrapersonal, interpersonal, organisational, community and public policy (see Figure 2) (24). What both the HF and SEF reminds us is that behaviours, and thus behaviour change, occurs in a complex, and often messy system. A dimension of change not often considered in many SEF is the time it takes across the layers as explored in the temporal extension of the socioecological framework (TESEF) (24). This acknowledges that facilitating behaviour change may involve all layers which may occur at different times and in disjointed ways.

### 4.2 Discussion of results

Participants injured in TP were more likely to be younger, male, snowboarders, and where more likely to be a day ticket user or a season pass holder, compared to those injured outside TPs, thus a distinct target group for injury mitigation and prevention strategies and communications strategies. However, as noted in the results, while most were statistically significant, small or weak effect sizes indicate that the differences may not be practically significant. Thus, while this discussion explore how we may address the differences, in practice, it may still be possible to consider most TP users as part of the broader snowsport cohort.

The diverse ticket types suggest they may be infrequent or new visitors (day tickets) or frequent visitors (season pass) (Table 3). This insight can help develop more precise personas for whom more targeted risk management strategies and messaging may be required to reach this cohort. For example, distinct ticket groups (day and multi-day) may be the result of a culture of wanting to get value for money and/or a difference in knowledge of the resort and the TP. In contrast, season pass holders may be more familiar with the resort, and potentially under less pressure to get value for money. Alternatively with the proliferation and popularity of multi-resort passes like Epic and Ikon (25), they could be also visitors unfamiliar with the resort. Thus, different messaging and modes of messaging may be required for infrequent and new visitors compared with regular visitors. This may include different social media and online messaging especially where day and multi-day tickets are bought online.

While alpine skiers had a higher helmet usage rate in TP (92%), they also reported a higher rate of head injuries than snowboarders. Increased helmet usage has not resulted in a correspondingly significant reduction in reported head injuries, though it is possible that higher reporting levels may be a result of the increased awareness of the potential sequelae of head injuries (4, 26). In contrast to previous research (22) higher skill levels of TP users did not have significantly different, nor lower, helmet usage than lower skill levels.

The potential risk of the use of alcohol within TP has been noted previously (2) while an increase in normal alcohol use when tourists are participating in snowsports has been observed in recreational participants and injured tourists (13, 27). Drug and alcohol as a potential contributing factor to injuries, while reported as low in TP, was twice that for outside TP. Breath testing of recreational participants for drug and alcohol may not be feasible, nor legal in some countries, however reinforcing the drug and alcohol messages of alpine responsibility codes could occur through limiting alcohol sale until later in the day.

TP injury patterns for skiers differs greatly from other resort areas where knee injuries may account for over 30% of skiers' injuries (3, 28, 29), compared with 13% reported within the TP. This may reflect the younger cohort in TP (28) as well as different terrain design (such as landing areas), speeds (30), and/or mechanisms of injury in TP (31).

As indicated by destination from base, TP injuries are more severe than non-TP injuries. However this may also be a result of the typical TP user as much as the risk of TP design or feature removal (1). For example, in this study 51.1% of TP injuries were minors (<18 years) compared with 38.5% outside the TP. Minors may be more frequently transported to hospitals out of a duty-of-care or an abundance-of-caution, while adults have been known to by-pass on-snow medical services in some cases (13).

### 4.3 Discussion of results in light of the three systemic models

Through the lens of the HoC, at the resort or organisational level, the easiest way to reduce TP injuries is to apply the first layer of the HoC, elimination (1), though this may not be desirable for business growth. After elimination is substitution or redesign (20) including materials redesign (32). This may mean changing features such as lower jumps, smaller gaps and grooming outrun and fall zones to decrease the injury risk as suggested by some (8, 14). The efficacy of these actions needs to be considered in light of participants' interaction with the environment especially the changeable natural environment (see also HF). Different administrative controls and communication strategies around signage may be required as two ticket types dominate here (day ticket and season pass). As seen in previous analysis (11), increasing use of PPE like helmets has not correlated with a decline in reported head injuries, however further data is required regarding feature type (14) and mechanism of injury to determine whether a helmet could have provided greater protection. As noted earlier, there may also the possibility that head injuries are reported more due to greater awareness of the long-term impacts of head injuries, especially concussion, but this has not been addressed in research so far.

From a HF perspective, snowsports occur in complex social systems that need to be considered when managing the risk of TP injuries (4). Thus, while TP feature removal or redesign may be part of the risk management, this does not address the business case for having TP nor the motivations of those who participate (2). Table 4 presents examples of modifiable and unmodifiable (5) components of the dynamic TP injury prevention “system” informed by this and previous HF research (Table 4).

**Table 4:** Human factors: examples of modifiable and non-modifiable risk factors.

	Modifiable	Non-modifiable
Environment	Pre visit:	Natural environment and elements e.g., the wind, visibility, snow conditions, and slope angles, altitude
	Online ticket sale and resort information	
	Marketing images and messages	
	TP design	
	During visit	
	TP maintenance	
	Information (online and in-place)	
	Resort signage: directional and Alpine Responsibility Codes	
People	Ticket type: day vs. season	Gender, age
	Behaviours and expectations	
	Culture of activity and/or resort	
Equipment	Activity: skiing, snowboarding	
	PPE: helmets, wrist guards, back guards	

**Figure 3:**
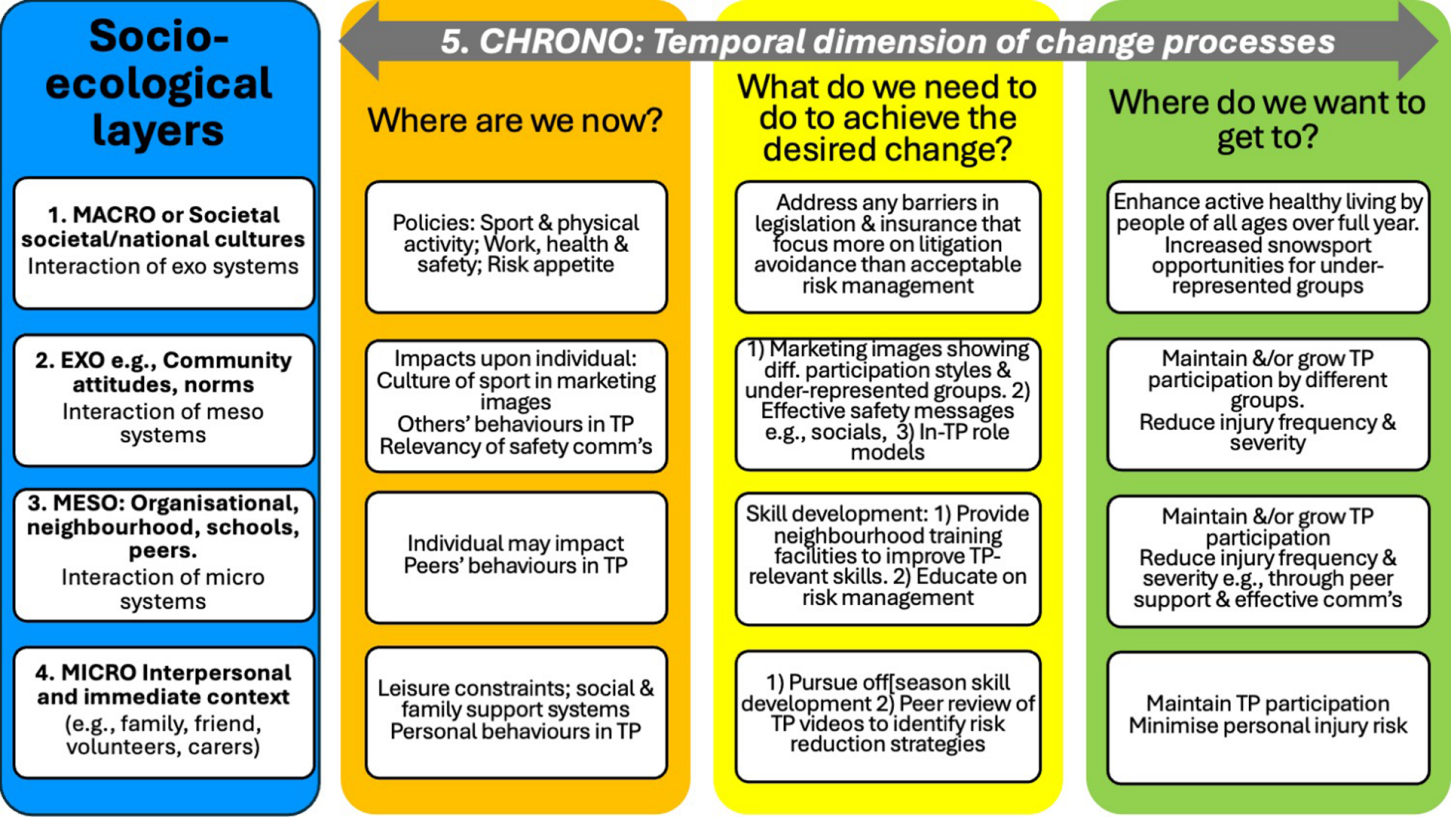
Example of TESEF applied to Terrain Park injury risk management. Source: the author.

The TESEF provides further insight into the complex social systems in which TP operate and the timeframes in which TP injury prevention needs to be considered. Figure 3 provides a TP-focused example drawing upon this and previous research (e.g., 2, 21, 31, 33, 34). As indicated, TP may be part of a broader health and well-being political agenda where there are many stakeholders both within the organization/resort (e.g., patrollers, marketers, shareholders) and beyond (e.g., families, public health).

Further, as noted above, understanding the motivations of participants, including sport and social motivations, will help guide risk management strategies. If young participants are motivated by hanging out with their friends and/or challenging themselves (2), then removing TP may result in shifting that behaviour to other resort and out of bounds areas with less risk management. While those seeking escape or freedom (2, 35) may resist additional restrictions. Whatever new strategies are applied, this will need to be evaluated to compare to pre-intervention injury risks, and to then inform future strategies.

### 4.4 Limitations

As this data is voluntarily collected by resorts and entered into a database, there may be variations in the data quality. However, this is offset by the benefit of being able to analyse a decade of data from 19 resorts. Another limitation is that the actual participation rates within TP vs. other resort areas is not known, nor easily obtained. As previously observed (2), the amount of time spent in a TP can vary greatly, e.g., 35%–100% of their time. Thus, to gather accurate exposure data would be costly and impractical, and thus the use here of the industry standard, macro level data, of participation, “skier visits”. Further, the resorts' skier visit data does not differentiate between activities e.g., skiing vs. snowboarding. More costly and limited observational methods would achieve this. Since this data was collected, individual resorts have increasingly retained management or their data, thus losing the opportunity to assess population-wide or multi-resort interventions.

## 5 Conclusion

TP are an important offering to tourists and visitors to resorts to attract and grow the sport, where effective risk management is to be applied within a complex multifactorial system. The injury reports presented here demonstrate the appeal to an often-younger, male audience, who tend to prefer snowboarding, than those participating outside the TP. Thus, they may present a clearer target group for communications, that resort-wide communications. If this group are not participating in the controlled environment of the TP, where else in the resort will they go to meet their personal and social goals?

From the frameworks explored, the hierarchy of controls may suggest elimination of TP or substitution, while human factors and socioecological perspectives provide guidance where TP are retained. Then, over time, the interactions between people, equipment and environment will be necessary to manage TP injury risks. Thus, reducing TP injury frequency and severity to acceptable levels, will occur concurrently with maintaining TP appeal to participants (2, 4). Any future interventions will then need to be evaluated to ensure their efficacy and inform future strategies.

## Data Availability

The data analyzed in this study is subject to the following licenses/restrictions: Access to data was made available under a commercial in confidence agreement that does not allow sharing with others outside the research team. Requests to access these datasets should be directed to office@cwsaa.org.
